# Women Health in Saudi Arabia: A review of non-communicable diseases and their risk factors

**Published:** 2014

**Authors:** AlJoharah M. AlQuaiz, Amna R Siddiqui, Riaz H Qureshi, Mona A Fouda, Maha A. AlMuneef, Fawzia A Habib, Iqbal M Turkistani

**Affiliations:** 1 AlJoharah M. AlQuaiz, Chair of Princess Nora Chair for Women’s Health Research, King Saud University, Riyadh, Saudi Arabia.; 2 Amna R Siddiqui, Chair of Princess Nora Chair for Women’s Health Research, King Saud University, Riyadh, Saudi Arabia.; 3 Riaz H Qureshi, Chair of Princess Nora Chair for Women’s Health Research, King Saud University, Riyadh, Saudi Arabia.; 4 Mona A Fouda, Chair of Princess Nora Chair for Women’s Health Research, King Saud University, Riyadh, Saudi Arabia.; 5 Maha A. AlMuneef, Chair of Princess Nora Chair for Women’s Health Research, King Saud University, Riyadh, Saudi Arabia.; 6 Fawzia A Habib, Chair of Princess Nora Chair for Women’s Health Research, King Saud University, Riyadh, Saudi Arabia.; 7 Iqbal M Turkistani, Chair of Princess Nora Chair for Women’s Health Research, King Saud University, Riyadh, Saudi Arabia.

**Keywords:** Women’s health, Non-communicable diseases, Risk factors, Saudi Arabia

## Abstract

This is a review of the changing pattern of chronic diseases among women in the Kingdom of Saudi Arabia (KSA). Data from national surveys conducted in KSA, whose results were published between 1996 and 2011 were used. The results showed that over a period of ten years the prevalence of obesity increased in Saudi women from 23.6% to 44.0% and in men from 14.2% to 26.2%; self-reported physical inactivity worsened in both women (from 84.7% to 98.1%) and men (from 43.3% to 93.9%); prevalence of smoking in women increased (from 0.9% to 7.6%), while it declined in men (from 21.0% to 18.7%). The prevalence of metabolic syndrome was significantly greater in women than men (42.0% versus 37.2%; p <0.01). In conclusion, Saudi women are potentially at a greater risk than a decade ago to develop cardiovascular diseases and diabetes mellitus**, **with a notable increase in obesity compared to men.

## BACKGROUND

Recent data from Western countries on trends in disease rates and risk factors among adult men and women have reported that risk factors are improving among men, while they are worsening among women.^1^^-^^4^ Women were previously considered to possess an advantage over men due to the protective effect of oestrogen against cardiovascular disease and its risk factors, and they were reported to have a longer life expectancy than men.^1^^,^^2^^,^^4^

However, the advantage of longevity in itself is not enough to infer that women are healthier than men. Currently, the well-being of women is generally affected by social influences, such as poverty, lower education and less pay than men, violence, male dominance, and lack of social equity and health care accessibility.^3^^,^^5^ On the other hand, higher smoking prevalence as well as occupational exposure and injury lead to higher prevalence rates of some major diseases among men than in women.^3^

Ministry of health in Saudi Arabia provides free health care to its nationals, its population is relatively younger and there are reports of increase in risk factors and chronic diseases over a period of less than a decade.^1^

Over the last thirty years Saudi Arabia has undergone socioeconomic development with improvements in health education, environment, urban migration and lifestyle; with decline in communicable diseases and increase in non communicable diseases.^1^

We, therefore, undertook a review of the published literature on non-communicable disease rates in recent years in Saudi Arabia to identify differences in disease rates and risk factors between men and women in this unique environment. To our knowledge, this review is the first attempt to examine the current status of women’s health in Saudi Arabia, while focusing on non-communicable diseases and their risk factors and compare their rates to those of Saudi men. 

## METHODS

This review used data from national surveys conducted in the KSA and published between 1996 and 2011.

***Data sources: ***These publications were identified using Medline database, PubMed Central, Google Scholar, WHO InfoBase, and references from retrieved articles. There were two major national surveys conducted one between 1990-1993 and another between 1995 – 2000. Studies based on these two surveys have pointed towards patterns and trends in chronic diseases and their risk factors. 

We searched Pub-Med with key words “Saudi Arabia” National; and then each key word as: diabetes mellitus, coronary heart disease, hypertension, dyslipidaemia, metabolic syndrome, obesity, physical inactivity, and smoking.

***Study selection: ***Exclusion criteria were for studies: not performed on a nationally representative sample, focused only on pregnancy, included age groups other than adults based on entirely, on treatment and practice guidelines when examining chronic diseases and their risk factors in relation to other co-morbidities than the ones of interest to this review; articles overlapping for the same chronic diseases in addition articles and in the same population and of the same authors; and finally studies published from language other than English had to be excluded; however, we did not find any study published in language other than English.

**Fig.1:**
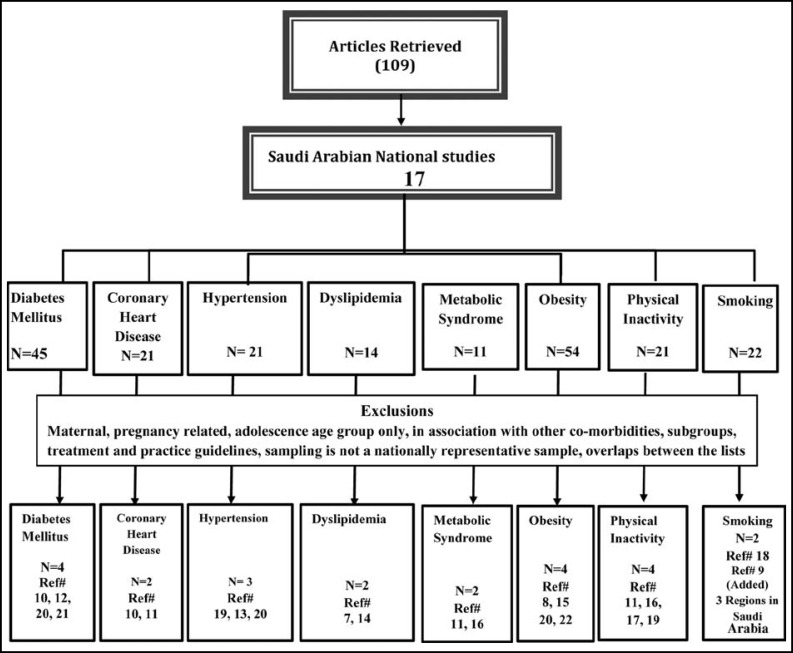
Extraction of 17 Saudi Arabian national studies and related articles satisfying inclusion and exclusion criteria

***Data extraction: ***The literature search was performed considering standards adopted as Moose guidelines for systematic reviews of observational studies.^6^ Two authors independently reviewed each article to determine if they should be excluded according to criteria; any differences in determination of exclusions were discussed and agreement reached for determination of inclusion or exclusion. The search yielded 109 articles (Fig.1), and a total of 17 articles were included. All studies were cross-sectional in design, included a nationally representative, community-based sample with multistage cluster sampling and determined the prevalence of risk factors and chronic diseases. Non-communicable disease rates were reported by sex. The national surveys and the published studies that were based on these surveys, along with enrolment periods (data collection), sampling techniques, sample size, age group of target population, diagnostic tools, and quality indicators that are specified in the study are provided in (Appendix-1).

All surveys were conducted under the auspices of the Saudi Arabian Ministry of Health (MOH). Data from other sources, such as national registries, government-issued statistic, and World Bank and United Nations reports were collected as indirect evidence. Small-scale studies conducted in Saudi Arabia, using data collected from certain women with specific conditions, were included when nationally representative data from community based samples were not available. Prevalence of disease or chronic disease risk factors among women were compared using data collected through these surveys. The temporal patterns of chronic diseases and their risk factors were compared between men and women. The major criteria considered in included studies were, title, research question, problem, sampling technique, distribution of risk factors or chronic diseases described by sex, assessment of confounding, statistical methods, and direction for future research.^6^

## RESULTS

Two national epidemiological surveys were conducted in Saudi Arabia, namely, the chronic metabolic diseases survey^7^^-^^10^, conducted between 1991 and 1993, and the Coronary Artery Disease in Saudis (CADiS)^11^^-^^19^ survey, conducted between 1995 and 2000. Additional reports that were based on a household screening survey on diabetes mellitus^20^^,^^21^, a national nutritional survey (1984–1991)^22^, a country-specific report of the STEP-wise Approach to Non-Communicable Diseases Surveillance, the WHO survey (2004)^23^, and the National Family Safety Program^24^ were also considered. Large-scale studies using standard tools and specific time periods as described above were included.

***Cardiovascular Disease: ***The prevalence of coronary heart disease (CHD), based on a nationwide representative sample from the 1995-2000 CADiS survey (n = 17,323), was 6.6% in men and 4.4% in women (p < 0.001) aged 30–70 years.^11^ This survey reviewed records of persons with angina or MI and the electrocardiogram diagnosis to estimate the CHD prevalence. CHD increased with age in both men and women. Waist circumference, systolic blood pressure, fasting blood sugar, triglycerides levels, and the history of current smokers were associated with CHD.^11^ Analysis of the World Health Organization (WHO) report (2004) on the global burden of disease worldwide indicated that cardiovascular disease (CVD) was the primary cause of death among women after menopause. This review showed that CVD accounted for 31.5% of the deaths due to CHD in women after menopause compared to 26.8% in men.

***Diabetes Mellitus: ***The study by Al-Nuaim^10^, a national representative survey conducted during 1990–1993 in Saudi Arabia, found that the prevalence of diabetes mellitus(NIDDM) differed significantly by sex; the NIDDM prevalence in urban areas was 11.8% among men and 12.8% among women (p < 0.001). Age, body mass index (BMI), and family history of NIDDM were the major risk factors for NIDDM.^10^ The CADiS study (1995–2000) reported that the prevalence of NIDDM among men was more than women (26.2% and 21.5%, respectively, p < 0.001).^12^ Reasons for these contradictory results could be explained by the age of the enrolled group (>15 years earlier in Al-Nuaim study versus > 30years in CADiS) and earlier WHO criteria used by former study. Also Al-Nuaim reported that 50% of the participants were newly diagnosed as diabetics compared to 27.9% in the Al-Nozha study; however, the ratio of women to men is unknown; nationally represented survey conducted after this did not report the burden of unmeasured diabetes mellitus in population. Also, the Al-Nozha study appeared to have better screening and diagnosis of the disease, which could have resulted in the higher reported rates. This is in accordance with another national survey (1991–1995) that reported the prevalence of NIDDM to be 17% in men and 12% in women for the >30-year age group (P<0.001).^21^ Old age was positively correlated with NIDDM among women. In the 50–59 years age group, more women than men were diabetic (34.3% versus 33.5%, respectively, p < 0.001).^12^

In collaboration with the WHO, the Saudi Arabian Ministry of Health established a Field Epidemiology Program, through which a national survey was conducted in 2004. For the age group of 25–64 years, the prevalence of NIDDM in men and women was reported to be 22.8% and 20.1%, respectively.^23^ It is evident that risk factors for NIDDM, particularly obesity and sedentary lifestyles that lack regular physical activity, are also rising in KSA (Table-I).

WHO reported in 2000 that Saudi Arabia was among the countries with the highest NIDDM rates and that NIDDM affected more Saudi Arabian men than women.^25^ Given the increasing burden of NIDDM in Saudi Arabia, improving the diagnostic and therapeutic services of the health care system is necessary.^12^

***Hypertension: ***Saudi Arabia has a young population; approximately half of the population was aged less than 18 years.^11^^,^^13^^,^^20^ A study by Nozha, using a high cut-off point, reported a systolic hypertension (HTN) prevalence (≥160 mm Hg) of 12.4% and of diastolic HTN (≥95 mm Hg) prevalence of 7.9% among individuals younger than 18 years of age; whereas prevalence of systolic and diastolic HTN was reported at 5.2% and 7.3%, respectively, among individuals aged 18–75 years. However, if cut-off was taken as 140/90, 20.4% suffered from systolic and 25.9% suffered from diastolic hypertension in 18 years and above age group. The same study reported a significant difference in systolic HTN between men and women, (11.0% in men versus 15.7% in women, p < 0.01).^13^

The overall prevalence of HTN was 7.3%, and it was not significantly different in men and women.^13^ Isolated systolic HTN (≥185 mm Hg), which is associated with a significantly higher risk of stroke and coronary heart disease (CHD) was common among older women.^13^ Furthermore, systolic and diastolic HTN are risk factors for fatal and non-fatal stroke as well as CHD.^13^ The CADiS study revealed a positive linear correlation between age and BP as well as between weight and HTN prevalence. The disturbing observation was that, despite using a high cut-off level to define HTN (≥160/95 mm Hg), nearly 67% of individuals were unaware that they had HTN.^13^

Another survey conducted between 1995 and 2000, which used a cut-off level of 140/90 mm Hg, estimated the HTN prevalence at 28.6% in men and 23.9% in women^19^(Table-I). A subsequent survey in 2004 reported similar rates, with systolic HTN prevalence (>140 mm Hg) of 29.0% in men and 21.0% in women.^23^

The only obvious difference between these two surveys is in the sample size; the CADiS survey had a sample of 17000 and WHO Stepwise survey had a sample size 5000 participants.

HTN is also associated with higher mortality rates among women than men, and rates of HTN are two- to three-fold higher among women who use oral contraceptives than among non-users.^26^ Compared to hypertensive men, women with high BP are more likely to develop left ventricular hypertrophy, diastolic dysfunction, and a steep age-related increase in arterial stiffness.^27^ Moreover, HTN plays a bigger role in the development of congestive heart failure in women than men.^28^ Therefore, enhancing HTN detection among women can also improve the detection of other CVD risk factors and reduce the risk of serious CVD.

***Dyslipidaemia and Metabolic Syndrome: ***A national household survey was conducted between 1990 and 1993 and included 4,539 Saudi Arabian individuals over 15 years of age.^10^ The results of this survey indicated that the measured serum total cholesterol concentration (TCC) was significantly higher for women than for men (4.2 mmol/l versus 4.0 mmol/l, p < 0.001). The prevalence of hypercholesterolemia (TCC, 5.2–6.2 mmol/l) was also higher, 9% for men and 11% for women,^7^ and the prevalence of hypercholesterolemia with TCC >6.2 mmol/l was estimated as 8% among women, compared to 7% among men. Hypercholesterolemia consistently increased with age and BMI, in both men and women, and it was significantly higher among normal weight women than men (p = 0.012). Given that the sample predominantly comprised a young population, it is noteworthy that a consistent increase in TCC was observed with increasing age in both sexes.

In a subsequent study conducted between 1995 and 2000 (n = 16,819) on individuals aged ≥30 years, the prevalence of hypercholesterolemia was 54.9% among men and 53.2% among women, exhibiting a >75% increase in prevalence from the previous survey (Table-I). Unadjusted prevalence rates of TCC ≥ 5.2 mmol/l were significantly higher in men than in women (p < 0.02) and was higher in those in the urban areas and with a higher education level (college-educated individuals) compared to those in the rural areas and those with a lower education level. The contradictory result between TCC values in men and women could be explained by the different age groups enrolled in the different studies. In the Al-Naim^7^ study, participants >15 years of age were enrolled compared to ≥ 30 years in the Al-Nozha study.^14^ Also, in the former study the largest difference in dyslipidaemia prevalence rate was observed among individuals aged <30 years.^7^

Hypertriglyceridemia, defined as triglyceride concentrations ≥ 1.69 mmol/l, had an overall prevalence of 40.3%, with higher rates among men than women (47.6% versus 33.7%, p ≤ 0.001).^14^

Hypertriglyceridemia and low levels of high-density lipoprotein (HDL) cholesterol levels are the main risk factors for ischemic heart disease (IHD) among middle-aged and older women; however, they cannot be used for predicting IHD in older men and have a less pronounced gradient among men in other age groups.^28^ The previously mentioned CADiS study also examined the metabolic syndrome in Saudi Arabia. The overall age-adjusted prevalence of metabolic syndrome was estimated as 39.3% (37.2% among men and 42% among women), with higher levels observed in urban areas. Low HDL levels affected 81.8% of women and 74.8% of men with metabolic syndrome.^16^

***Obesity: ***The national survey conducted in Saudi Arabia from 1991 to 1993, included a population (n = 13,177) over 15 years of age.^10^ BMI was significantly higher among women than men (24% versus 16%, respectively) across all age groups, increased with age in both sexes, and peaked during the fifth decade of life. Similar values were reported by more recent surveys done in KSA.^8^^,^^15^^,^^20^^,^^22^^,^^23^ Overweight percentage was higher among men (27.23%) than that in women (25.20%) across all geographic regions and surveys in various time periods; similar results were reported by other national studies in Saudi Arabia.^8^^,^^15^^,^^20^^,^^22^^,^^23^ Overweight and obesity were more prevalent among illiterate and high income individuals who resided in Saudi urban neighbourhoods in early 1990s.^8^ The obesity rates in Saudi Arabia were among the highest, based on the WHO criteria, and fell above the 85^th^ percentile of obesity in the US population aged 20–24 years.^8^

The subsequent national survey, conducted in the KSA between 1995 and 2000 (n = 17,232) among selected households, reported a prevalence of overweight of 36.9% (BMI range: 25.1–29.9 kg/m^2^) that was significantly higher among men (42.4%) than that among women (31.8%). However, significantly more women were obese: a BMI ≥ 30kg/m^2^ was observed in 44% of women and 26.4% of men (p < 0.001). Obesity was also more prevalent among illiterate individuals, high income groups, and urban residents. However, obesity was highest in the 40–49 year age group in both sexes.^15^ The prevalence rates for overweight, obesity, and severe obesity among Saudi Arabian adolescents (2010 census) aged 13–18 years were 26.6%, 10.6%, and 2.4%, respectively.^29^

***Physical Inactivity: ***Physical inactivity increased by more than two-fold among men in Saudi Arabia within the last two decades (Table-I), whereas it has long been the predominant lifestyle among Saudi women. Therefore, extreme efforts are required to modify this rooted lifestyle among women because it constitutes a major risk factor for chronic disease.^3^^,^^17^ The CADiS study revealed an overall prevalence of physical inactivity of 96.1%, with more women inactive than men (98.1% versus 93.9%, p < 0.001).^17^ Physical inactivity increased with age, especially among men, and decreased with higher educational levels. Physically active individuals had lower BMIs and reduced waist circumferences.^17^ The WHO reported that 60%–85% of adults around the world are not active enough to achieve the benefits of physical activity.^30^ In a study conducted in Riyadh city the most frequently reported obstacle to physical activity was lack of resources (80.5%), which was most widely reported among women and low income individuals.^31^ This high prevalence of inactivity, accompanied by a high-calorie diet intake, has led to the development of global overweight and obesity, type 2 DM, and CHD epidemics in recent years.^30^

***Smoking: ***Prevalence of smoking among Saudi Arabian women is reported to be much lower than that among men; however, their rates have been increasing in both sexes. A study published in 1999, conducted with 8,310 individuals aged ≥15 years who were randomly selected from the three regions of five regions (Northern, Western, southern, Eastern, and Central) of Saudi Arabia because of incomplete data obtained from two regions—namely, the northern region (in which smoking was recorded for 27 people) and the central region (in which there was no recording for smoking)—they were excluded from the analysis.. This study reported the prevalence of current smoking at 21.1% among men and 0.9% among women.^9^ Another national study, conducted between 1995 and 2000, reported the prevalence of current smoking at 12.8% (18.7% among men and 7.6% among women),^18^ while the WHO field study in Saudi Arabia reported it at 36% among men and 6% among women. The increase in smoking prevalence rate was associated with lower socioeconomic status, lower education levels, divorce, and certain occupations such as military and self-employment jobs.^32^

The prevalence of smoking among female university students (n = 1,050) was observed to be 11%, 44% of which were using water-pipes, 36% were cigarette smoking, and 20% were using both.^33^ Another study (n = 7,550) conducted with undergraduate students at the King Saud University (KSU) in Riyadh, showed an overall smoking prevalence of 14.5% (32.7% among male students and 5.9% among female students).^34^

Smoking prevalence among women varies significantly across countries, such that the percentages range from an estimated 0.4% to 39%.^35^ Although the rate of current smoking among Saudi Arabian women is increasing, it is still lower than the rates observed among European and American women.^35^

## DISCUSSION

The sampling technique to represent the Saudi population estimates is a major strength of the studies included in this review. These surveys (1990-93 and 1995-2000) provided direction to health care system provided by the ministry of health to address non communicable diseases.^1^ Our review revealed a consistently higher prevalence of overweight among men than that among women; however, all reports showed constantly higher obesity prevalence rates among women than men. The45% increase in obesity among men and women of the Saudi Arabian population over a period of approximately a decade and a half, is comparable to the increase in the overall prevalence reported in the US in both sexes for the 20–70-year age group in the NHANES data (1976–2000).^5^ The prevalence of obesity in the US increased from 15.0% in 1976–1980 to 30.9% in 1999–2000, with no significant overall difference between men and women; however, a difference was observed among population subgroups according to age, race, and ethnicity.^36^

The rapid changes in lifestyle within the Saudi Arabian population are apparent, with currently three-quarters of adult women being either overweight or obese, compared to two-thirds of adult men. Our results further revealed a positive association between age and hypercholesterolemia, BMI (obesity), hypertriglyceridemia, NIDDM, and HTN. Obesity prevention in the Saudi Arabian society requires intervention among the younger age groups to reduce the spread of this multi-factorial chronic disease risk factor, as a rising trend is evident among children and adolescents.^29^

**Table-I:** Comparison of health characteristics in Saudi Arabian women and men by time period

*Health indicators in Saudi Arabia over the decades*			
*Year Published *	*1970 – 79*	*1990-1999*	*2000-09*
Life expectancy (yrs)^39^	53.8	69.0	74.9
Literacy in women (%)^40^^,^^41^	16.4	50.0	71.0
IMR* (/1000 LB)^39^	65.0	36	15.0
Annual mortality rate under age 5 years, 1990 (43/) 2009 (21)^39^	179	45	26
MMR** (/100000 LB)^42^	48.0		14.6
Crude Birth Rate^39^^b^		47	22
*Prevalence of Non Communicable diseases and risk factors in Saudi Arabia*			
*Disease risk - Publication year *		*1990 – 99 *	*2000 – 09 *
*Coronary artery disease* ^11^ Men (30-70 years) Women (30-70 years)			6.6% 4.4%
*Systolic hypertension / diastolic hypertension,* ^13^^,^^20^ Men (30-74 yrs; >160/95 mm Hg )^a^ (30-70 yrs>140/90 mm Hg)^b^ Women (30-74 yrs; >160/95 mm Hg)^a^ (30-70 yrs; >140/90 mm Hg)^b^		^a^6.69 % / 11.4% ^a^ 9.1% / 11.4%	^b^28.6% ^b^23.9%
*Isolated systolic hypertension* ^13^^, ^^19^ Men Women		1.4 % 2.0 %	5.7 % 5.0%
*Diabetes Mellitus* ^10^^, ^^20^^,^^21^^, ^^12^ Men Women		9.7 % 7.0 %	26.2% 21.5%
*Metabolic Syndrome* ^16^ Men Women			37.2% 42.0%
*Elevated Serum Cholesterol* ^7^^,^^10^^, ^^15^^, ^^16^ Men (Age > 15 &> 30 years 1990-9 to 2000-09 respectively) Women (Age > 15 &> 30 years 1990-9 to 2000-09 respectively)		9% 11%	54.9% 53.2%
*Obesity (BMI >30kg/m2)* ^8^^,^^10^^, ^^17^^, ^^18^^,^^20^ Men (Age > 15 &> 30 years 1990-9 to 2000-09 respectively) Women (Age > 15 &> 30 years 1990-9 to 2000-09 respectively)		13.05 - 16.0% 20.26 - 24.0%	26.4% 44.0%
*Overweight (BMI =25-29.9kg/m2)* ^8^^, ^^10^^, ^^17^^, ^^18^^, ^^20^ Men (Age > 15 &> 30 years 1990-9 to 2000-09 respectively) Women (Age > 15 &> 30 years 1990-9 to 2000-09 respectively)		27.23 % 25.20 %	42.4% 31.8%
*Self-reported Physical Inactivity* ^17^^, ^^20^ Men Women		43.3% 84.7%	93.9% 98.1%
*Smoking* ^9^^, ^^18^ Men Women		21% 0.9%	18.7% 7.6%
*Prevalence of Non Communicable Diseases and risk factors in other settings *			
*Obesity (NHANES* *** * – USA)* ^3^^,^^4^ Men Women		20.9% 27.0 %	30.4% 34.1%
*Coronary artery disease (NHANES –USA)* ^3^ Men (35-54Years) Women(35-54Years)		2.5% 0.7%	2.2% 1.0%
*Mean Framingham Coronary Risk Score-USA* ^3^ Men Women		8.6 3.0	8.1 3.3
*Stroke prevalence (NHANES-USA)* ^3^ Men (45-54 years) Women (45-54 years)		1.68 1.08	1.04 2.54
*Mean BMI* § * Sweden* ^43^ Men (40-60 YEARS) Women (40-60 YEARS)		25.9 25.2	26.8 25.9

IMR* : Infant mortality rate,

MMR** : Maternal mortality rate

NHANES***: : National Health and Nutritional Examination Survey,

BMI§ : Body Mass Index.

**Appendix-1:** Major Surveys and their publications in Saudi Arabia on chronic diseases and risk factors

		*Disease & risk factor burden*	*Sample* *Size (N)*	*Age*	*Sampling* *Method*	*Diagnostic tests & measurements*	*Agency*	*Quality*
1	Al-Nuaim^7^^-^^8^^, ^^10^ (National Chronic Metabolic Diseases Survey; 1990-93)	Chronic diseases^10^	13177	15 + year	-Multistage, stratified cluster; & probability Proportional Sampling National Level	-Height, Weight -Blood for total cholesterol - Blood Glucose and -Glucose tolerance test	MOH*	-Weighted sample -Study Tools done by doctors -Blood tests using standard criteria
		Hypercholesterolemia^7^	4539					
		Overweight and Obesity^8^	13177					
2	Al-Nozha^11^^-^^19^ (Coronary Artery Disease in Saudis (CADiS); 1995-2000)	-Coronary heart disease^11^ -Obesity^15^	17232	30- 70 years	Two stage stratified cluster sampling Stratification based on rural urban areas National Level	-Height, Weight -Waist circumference -Blood Pressure -ECG -Questionnaire -Clinical Exam -Fasting sugar -Lipoproteins - High density -Low Density -Smoking prevalence	KACST§ MOH	-Validated Questionnaire -Complete Physical Examination -ECG &Laboratory measurements standard criteria -Current, passive. Ex smoking;, type, quantity, duration
		Hypertension ^13^^,^^19^	17892					
		Diabetes Mellitus^12^^,^^14^ Hypercholesterolemia	16819					
		-Physical Inactivity^16^ -Metabolic Syndrome^17^	17293					
		Smoking^18^	17350					
3	El-Hazmi^20^^-^^21^ (A study of Diabetes Mellitus in Saudis. Project AT-MW-10; 1991-95)	Diabetes Mellitus^21^	25657	> 2 years	Household Screening & nutritional survey for DM & Lipids National Level	-Fasting Blood Glucose -Lipid profile -Weight -Height	KACST KSU^ MOH	Extensive laboratory and survey methods Used standardized methods
		Diabetes Mellitus: multi-factorial disorder^20^	14660	>14 years				
4	WHO^23^ Stepwise Approach to NCD surveillance Field Epidemiology Survey 2004	Non-Communicable Disease Surveillance Country Specific Report-2005^23^	5000	15-64 years	Cross-sectional community-based study. Multistage age stratified Cluster sampling National level	Questionnaires; behavioral risks physical exam (BMI), biochemical tests for blood sugar and lipid profile	WHO! FETP¥ MOH	-Standard Survey and Laboratory methods -Double data entry

NCDS* : National Chronic Metabolic Disease Survey

MOH** : Ministry of health

CADiS***: : Coronary Artery Disease in Saudis

**KACST§:** : King Abdulaziz City for Science and Technology

KSU^ : King Saud University;

**WHO-FETP¥:** : World Health Organization- Field Epidemiology Training Program.

The Saudi data available currently indicate that chronic diseases are more prevalent in men than in women. CHD, NIDDM, HTN, and other chronic disease risk factors occur also more frequently in men than in women. Obesity, a major risk factor for all such health outcomes, indicated by a BMI ≥ 30 kg/m^2^, was observed in three-quarters of adult women compared to two-thirds of men. Other risk factors, such as smoking and physical inactivity, were mainly observed at higher rates among the low socioeconomic groups. A rise in smoking rates among younger women could lead to further increases in unfavourable chronic disease outcomes among women in the future. Health education and other preventive efforts should be undertaken to inform Saudi Arabian women about the importance of modifying their lifestyle to reduce obesity and waist circumference, to quit smoking and increase their physical activity.

Despite possible differences from trends observed in other countries, risk factors and chronic disease burden have followed a rising trend in KSA in the last two decades. Moreover, a large proportion of the population are unaware of their health status, especially regarding NIDDM.^7^^,^^8^^,^^11^^,^^13^^,^^17^^,^^18^^,^^20^ Clearly, NIDDM has become an epidemic with a prevalence increase from 7% to 22% observed among women, such that a greater proportion of women were diabetic than were men in the 50–59-year age group.^11^ A recent published review on NIDDM also highlighted in detail the alarming increase in the prevalence of NIDDM in Saudi Arabia.^37^

The reports on systolic and diastolic HTN (12.4% and 9.9%, respectively) in the Saudi younger population (<18 years of age)^13^ should be explored within this setting, and findings should be verified through longitudinal or follow-up studies. The prevalence rates of systolic HTN (age > 60 years), obesity, metabolic syndrome, and physical inactivity were higher among Saudi Arabian women than that among men. The prevalence of risk factors for CVD were extremely high among middle-aged and older women. Therefore, it is critical to educate women and health care providers on CVD risk factors and possible methods to control them. In 1996, the probability of developing coronary artery disease (CAD) among women aged 30–70 years was estimated at 50%, calculated using the computer model based on the Framingham Heart Study in USA.^4^ The results of that study showed high rates of hypercholesterolemia (31%), NIDDM (30%), HTN (13.8%), family history of CAD (6%), and obesity (45%). The estimated rates for the probability of developing CAD within 5, 10, and 12 years were 4.31%, 9.88%, and 12.25%, respectively. Our review provides evidence consistent with the probability model developed 15 years ago. The rising trend of stroke rates among women in the US should be considered a warning, because stroke rates may potentially follow a similar trend in Saudi Arabia, if the high prevalence of risk factors among women are not addressed through preventive efforts and health education.^5^

In KSA, smoking prevalence varies among women and is most prevalent among younger, unmarried women, who are more likely to use water-pipes, as they assume that this form of smoking is less harmful.^35^ Thus, awareness campaigns should be directed at the younger age groups to emphasize the hazards of smoking, and they should correct the misconception that water-pipe smoking is a form of ‘safe smoking’.

Given the temporal changes in Western countries, efforts should be made to enhance diagnostic and therapeutic tools in countries that are experiencing increasing rates of CVD. Additionally, efforts should be undertaken to increase awareness among health care workers in Saudi Arabia to address the changing needs in women’s health during the various stages of life, especially as screening algorithms and criteria could differ between men and women of different age groups and diverse risk factors. Additionally, studies targeting the health system should be conducted in Saudi Arabia in order to evaluate women’s accessibility to health care services, along with assessing their preventive health-seeking behaviours. Given that more Saudi Arabian women are obese than men, it is important to increase awareness of abdominal obesity as an independent risk factor for CVD among women.

Despite remarkable improvements in literacy rates, infant and maternal mortality, and life expectancy among Saudi Arabian women over the last 40 years, the life expectancy of women is still lower than that observed in many developed countries (Japan = 86, Australia = 84, UK = 82, USA = 81, UAE = 80, KSA = 75 years).^38^ It is vital to promote healthy lifestyles that include regular physical activity and dietary modifications, involving lower intake of fatty foods and sugar, and higher intake of fiber, fruits, vegetables, vitamin D, and calcium (including supplementary). Maintaining a healthy body weight and avoiding overweight and obesity should be the focus of preventive and educational programs, including media programs. Increasing women’s accessibility to exercise facilities, providing safe walking areas for women and encouraging their use are likely to help reduce weight and disease risk. Smoking prevention and cessation is also important, given the rise in smoking rates among the younger population of Saudi Arabian women. These risk factors should be addressed at a national and regional level, with the aim of early identification, prevention, and treatment.

The strength of this review is based on the data that it evaluated, which was collected using population-based surveys representative of the Saudi Arabian population. The two major surveys used sound scientific methodology and established standardized tools. However, the data used in these surveys was cross-sectional in nature, such that disease and disease risk factors were studied concurrently so that the temporal relations between risk factors and disease could not be definitively established.

Factors affecting or causing the significant rise in non-communicable disease risk factors in this population should also be further explored within the context of the evolving large gene pool, given the high consanguinity rates of this population.^1^ Future studies using models that depict the influence of perceived barriers and self-efficacy on lifestyle changes should also be conducted.

## CONCLUSION

Saudi Arabian women have experienced a significant rising trend in obesity, which is likely for making them more highly susceptible to developing CVD and DM than Saudi Arabian men. Appropriate measures to improve women’s health include effectively promoting healthy lifestyles that involve regular physical activity. These efforts should be given priority and should be implemented through all possible means, including the primary health care services.
